# PB.34. Marker clip placement may not be needed in breast cancer patients undergoing neoadjuvant chemotherapy with a view to breast-conserving surgery whose tumours are associated with microcalcification

**DOI:** 10.1186/bcr3702

**Published:** 2014-11-03

**Authors:** M Sreenivas

**Affiliations:** 1 UHCW Hospitals NHS Trust, Coventry, UK

## Introduction

Patients who are potential candidates for breast conservation surgery following neoadjuvant chemotherapy have their tumours marked with a maker clip to guide surgery. Some of the breast cancers are associated with microcalcification. So far we have not seen any publication showing the potential use of tumour-associated microcalcification, which could be used as marker of the tumour bed instead of a marker clip. We present prospective case series where microcalcification associated with tumour had been used to guide surgery

## Methods

Thirty-eight patients with breast cancers underwent neoadjuvant chemotherapy between 1 December 2012 and 7 May 2014 at a single institute. At the end of chemotherapy 16 patients underwent successful breast-conserving surgery (BCS). Nineteen patients' tumours were associated with microcalcification.

## Results

Of the patients who underwent successful BCS, seven had tumour-associated microcalcification. In six of these patients the tumour-associated microcalcification was solely used as a marker of the tumour bed. In one case, due to the presence of few tiny eccentric specks of microcalcification associated with the tumour, it was decided to place a maker clip. In all seven cases, tumour-associated microcalcification was readily apparent during localisation.

## Conclusion

Traditionally, placement of commercially available markers clips (costs around £55 per clip) is recommended for patients considered suitable for BCS following neoadjuvant chemotherapy. Albeit a small series, our experience demonstrates that tumour-associated microcalcification will still be apparent after completion of neoadjuvant chemotherapy and as such can be used as a marker of the tumour bed, thus saving costs associated with clip placement.

